# Testosterone in patients with borderline personality disorder: from hair analyses to social behavior

**DOI:** 10.1016/j.cpnec.2026.100371

**Published:** 2026-07-27

**Authors:** Katja Wingenfeld, Eugenia Kulakova

## Abstract

Clemens Kirschbaum provided the important inspiration for our team to extend the assessment of steroid hormones beyond blood and saliva samples to include hair analyses. This enabled the investigation of the effects of long-term, cumulative steroid hormone secretion. This novel methodological perspective provided particularly compelling for the study of long-lasting stress-related mental disorders, such as borderline personality disorder (BPD). In an initial study, we found unaltered hair cortisol but increased hair testosterone in women with BPD. Notably, the latter finding inspired us to further investigate the role of testosterone in this patient population. Across subsequent studies, we replicated the finding of increased basal testosterone in a large sample of patients with BPD and identified associations with prosocial behavior and symptom burden, particularly internalizing symptoms. The present paper reviews these and related findings of testosterone in BPD and discusses their clinical relevance and implications for further research.

## Highlights

• The present paper reviews findings of testosterone in BPD and discusses their clinical relevance and implications for further research.

## 1 Why investigating testosterone in borderline personality disorder?

Testosterone is an important sex hormone present in both sexes. In females, its concentrations are lower than in males, and it is produced by the ovaries and the adrenal glands. Like other neurosteroids, testosterone can also be synthesized within the nervous system [1]. The initial idea to investigate testosterone in borderline personality disorder (BPD) stemmed from three lines of observation. First, neuroendocrinology largely agrees that different hormone systems are closely associated and interconnected. While the hypothalamus pituitary adrenal (HPA) axis has received a lot of attention, the hypothalamic pituitary gonadal (HPG) axis has been largely neglected, especially in BPD. Second, in clinical psychiatric research testosterone has been repeatedly linked to affective symptoms, like depressive mood and anxiety, and showed effects on cognition and social behavior. Thirdly, on the level of reproductive endocrinology, women with BPD are frequently observed to suffer from polycystic ovary syndrome (PCOS), a hormonal disorder associated with elevated androgen levels. This overlap suggests that hormonal imbalances linked to testosterone, particularly those influencing mood and reproductive function, may contribute to the symptomatology of BPD.

A large amount of studies investigated the function of the hypothalamus pituitary adrenal (HPA) axis in stress-related mental disorders such as major depressive disorder (MDD), post-traumatic stress disorder (PTSD) and borderline personality disorder (BPD). Findings suggest partly overlapping and partly disorder-specific patterns of HPA axis function, as well associations with cognitive functions within disorders [[2], [3], [4]]. In BPD, meta-analytic evidence suggests enhanced basal cortisol release and blunted cortisol reactivity to psychosocial stress [5,6]. Stress and cortisol then effect cognition, and can impact and impair learning, memory formation [7], and social cognition [4]. In addition to an activation of the HPA axis during stress, several studies demonstrate stress-associated testosterone increase [8,9], particularly in the Trier Social Stress Test (TSST) [10]. Importantly, while the reciprocal relationship between HPA and HPG axis hormones is undisputed, its exact mechanisms are only incompletely understood [11]. Activation of the HPA axis can have both, suppressing and stimulatory effects on HPG axis function. Sex hormones like testosterone and estradiol can mediate the stress response via influencing glucocorticoid secretion and feedback regulation. Testosterone, in contrast to estrogens, has been shown to inhibit HPA axis function [12]. Importantly, the behavioral effects of testosterone often depend on cortisol concentrations. This non-linear interaction between both axes has been acknowledged by the dual-hormone hypothesis [13].

In clinical studies, testosterone levels have been repeatedly related to depressive mood states and anxiety, with most studies suggesting that low testosterone levels increase the risk of these mental disorders [14]. In this context, elderly men have been investigated most frequently, suggesting that in this group hypogonadism (i.e., lowered production of gonadal hormones, particularly testosterone) was positively associated with psychological complaints [15]. However, in a large cohort study, lower testosterone was primarily associated with mental disorders in women [14], and evidence shows higher levels of testosterone in women with severe MDD [16]. Taken together, while the influence of testosterone on mental health is strongly supported, neither the direction nor the exact mechanism of this influence is yet clear, as important factors like sex and age, as well as their interaction, have not received systematic investigation.

With respect to testosterone effects on cognition, animal studies show positive effects of testosterone on hippocampal activity and cognition function. Additionally, testosterone seems to influence social recognition and social learning and promotes social behavior such as aggressive behavior to reach higher status [1,17]. Findings in humans are less clear. Here, the effects of testosterone on hippocampal volume and episodic memory seem to interact with cortisol levels [18]: only in a state of high cortisol did testosterone show positive correlations with hippocampus volume and memory performance. In social animals like humans, the effects of sex hormones on behavior are of particular interest, as central interpersonal motives such as aggression, dominance, social approach behavior, and reward seeking have been related to testosterone levels [1,17]. However, in humans, the relationship between testosterone and status is more complex than in other animals, as social status can also be achieved via prosocial and altruistic behavior [17]. Social cognition and behavior play an important role in humans for successful interactions and to establish interpersonal relationships. While several mental disorders are characterized by problems in interpersonal functioning [19], it might be the case that BPD, with key symptoms including unstable relationships, interpersonal aggression and fear of abandonment, represents a form of interpersonal dysregulation and instability that might be associated with cognitive and affective functions under the influence of testosterone.

Patients with BPD further show a high rate of somatic complaints and comorbidities, such as obesity, type 2 diabetes, cardiovascular disease and, consequently, metabolic syndrome [20]. This is also the case in patients with PCOS [21]. Accordingly, it is not surprising that women with BPD show an enhanced prevalence of PCOS [22]. Roepke and colleagues found higher free testosterone, androstenedione and 17a-hydroxyprogesterone concentrations in blood serum of women with BPD compared to healthy women. The prevalence of PCOS was 30.4% in the patient group compared to 6.9% in the control group. Taken together, this suggests an increased prevalence of hyperandrogenism in women with BPD [23].

In sum, several lines of research suggest that a focus on investigating testosterone and its association with psychological symptoms and cognitive processes might be fruitful in BPD. To follow this lead, we initially set out with the expectation that 1) patients with BPD would show higher levels of testosterone compared to healthy individuals and that 2) testosterone levels might be associated with aggressive behavior and externalizing symptoms.

## 2 Basal testosterone in BPD

Evidence of enhanced testosterone secretion in patients with BPD started to emerge about a decade ago [24,25]. One study compared testosterone in 55 patients with BPD (35 women) and 47 healthy controls (26 women) and found higher testosterone levels in the patient group. Naturally, males presented higher levels than females but there was no interaction between sex and group [24]. Additionally, an elevated cortisol awakening response (CAR) was found only in female patients with BPD. In this study, testosterone was measured in saliva at two measurement points in the same afternoon. In our own subsequent study, we decided – as initially inspired by Clemens Kirschbaum – to use a cumulative measure of steroids in hair samples [25]. We investigated 18 women with BPD and 17 healthy women and found increased hair testosterone levels among BPD patients compared to controls, suggesting that levels were increased long-term, leading to cumulative effects as measured by hair samples. In contrast, no differences between BPD patients and healthy women regarding hair cortisol levels emerged. These observations fit to the abovementioned finding by Roepke and colleagues, who reported higher testosterone (measured in two fasting serum blood samples) in women with BPD compared to healthy women [23]. In the sample of Roepke, women with BPD also showed higher PCOS prevalence. Higher testosterone concentration is an etiological feature of PCOS, and also points towards altered long-term testosterone secretion in these patients.

**Fig. 1:**
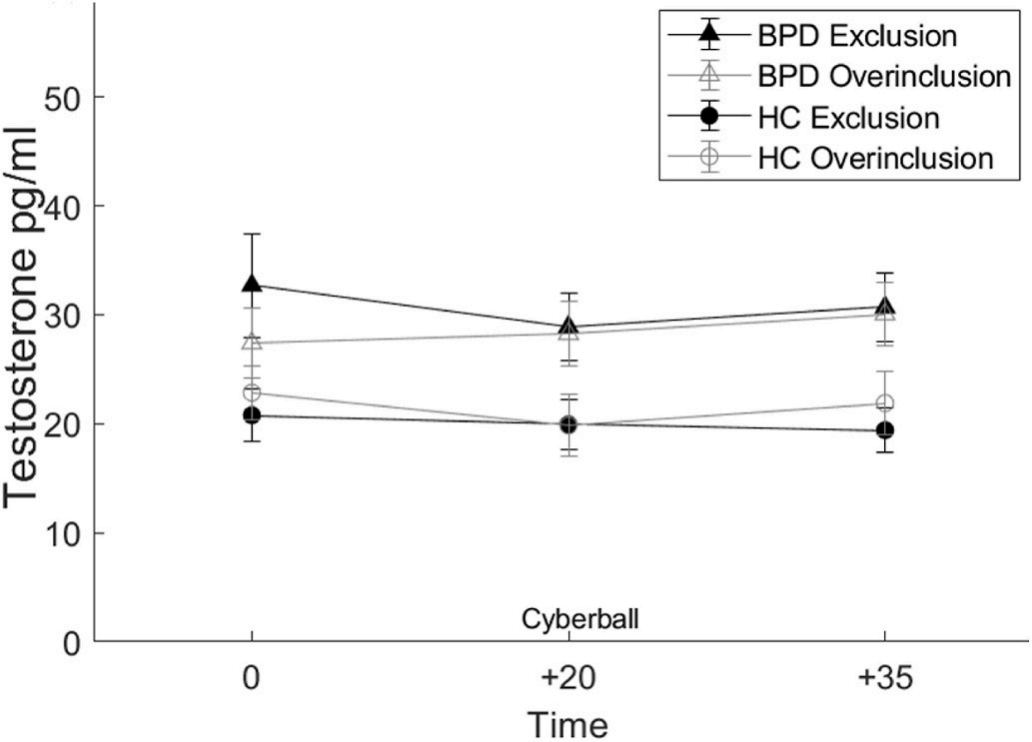
Testosterone values in individual with BPD and healthy controls. Figure adapted from Graumann et al., 2025.

In a more recent study, we aimed to replicate the finding of enhanced testosterone levels in BPD in a larger sample. We recruited 98 women with BPD and an equal amount of heathy controls and collected three saliva samples within one afternoon. As expected, we found higher testosterone levels in the BPD group [26], see Fig. 1). Consistently, another recent study by Bonfig et al. reports higher basal testosterone levels in mothers with BPD (N = 27) compared to healthy mothers [27].

In sum, there is growing consensus that patients with BPD show elevated testosterone levels, suggesting HPG axis related disturbances. Importantly, these results have been particularly consistent in female samples, while systematic sex-dependent investigations in large mixed or male samples are still missing. Nevertheless, these findings raise the question whether and how HPG alterations that are associated with increased testosterone levels are related to affective, cognitive and behavioral problems reported in this population.

## 3 Testosterone response to stress in BPD

Testosterone shows a diurnal variation with highest values in the morning [28]. In females, this diurnal course seems to be less pronounced. Furthermore, while variation during the menstrual cycle shows increased testosterone levels around ovulation, large inter-individual variability in cyclicity exists [29]. On top of these fluctuations, testosterone secretion varies due to external demands such as stress, although not all findings are consistent. In one of our own studies, we found testosterone to increase after psychosocial stress induced with the TSST in contrast to a non-stressful control condition, but there were no differences between healthy individuals and patients with BPD [9]. This result is in line with the so-called “challenge hypothesis” which states that a testosterone increases in anticipation of a challenge or competition [30,31]. Of note, the TSST is a social-evaluative stressor which can be characterized by posing a challenge to the social self, including one's social evaluation and reputation. According to the “biosocial model of status” [32], it is plausible that testosterone rises in situations when the individual is forced to show assertive or dominant behavior to maintain or protect social status. A different kind of psychosocial stress is social exclusion, which threatens the basic need of belonging that is central to humans and other social species. It is well known that patients with BPD are more sensitive to social rejection and negative evaluation and show higher expectations to be rejected across situations [33,34]. Interestingly, one study revealed a decrease in testosterone in healthy women after social exclusion induced with the Cyber-ball task - an experimental procedure which is frequently used to provoke social exclusion [35]. In our own study using the Cyberball game, we did not see such an effect. Instead, testosterone levels remained unchanged throughout social exclusion as well as the control condition of over-inclusion (see also Fig. 1) [26]. In summary, while research on testosterone's role in stress responses, especially in BPD, is still emerging, available evidence suggests that BPD may be characterized by a unique pattern of endocrine responses to stress. While meta-analytic data supports a blunted cortisol reactivity in response to psychosocial stress [5], alterations in the HPG axis remain less clear. Nonetheless, the pattern of enhanced testosterone secretion alongside reduced HPA axis activation fits with the dual-hormone hypothesis, which posits a complex, bidirectional interaction between both axes. It seems likely that this interplay influences stress reactivity and behavioral outcomes in BPD.

## 4 Testosterone and (social) behavior in BPD

As mentioned above, patients with BPD are highly susceptible to social rejection and exclusion. Moreover, the disorder is characterized not only by emotional instability and impulsivity, but also by a pervasive pattern of instability in interpersonal relationships and fear of abandonment [19]. The latter seems to be relatively stable over time, while other symptoms such as anger, impulsivity and dissociative symptoms worsen under stress [4,36]. This invites the question whether steroid hormones, particularly testosterone, are associated with (stress associated) behavior and social cognition in BPD. However, up to now, only few studies have addressed this topic.

One study compared mothers with BPD with healthy mothers during free-play with their children and rated maternal, child and dyadic behavior [27]. Mothers with BPD showed more negative states within the dyad, which was mediated by higher basal testosterone levels and cortisol reactivity. Interestingly, in another study by Rausch and colleagues, no association between basal testosterone and self-reported anger and aggressiveness was found in BPD, while overall testosterone levels were enhanced compared to healthy individuals [24]. Instead, there was a positive correlation between negative emotions and the cortisol awakening response, which was enhanced in the female subsample of BPD patients. In another study, an approach-avoidance task with facial stimuli was performed during functional MRI measurement [37]. Patients showed deficits in emotional action control with faster approach versus avoidance responses to angry faces. However, no association between behavioral and hormonal measures was observed. In a recent study from our lab, we used a different approach: we induced stress via social exclusion with the Cyberball game, and measured (pro-) social behavior by employing the “dictator-game”, measuring sharing behavior [26]. Interestingly, patients with BPD shared more generously than healthy individuals. The percentage of shared money further correlated positively with baseline testosterone, which at first seems at odds with the assumption that testosterone is positively associated with status seeking and aggression. Punishment behavior, however, captured with the “ultimatum game”, did not correlate with testosterone. This finding might be explained with reference to the “Biosocial Model of Status” [32], which suggests that high status (possibly accompanied with high testosterone levels) can be reached through prosocial or altruistic behavior [17,38].

Taken together, the relationship between testosterone and prosocial behavior in BPD is not as straightforward as initially assumed and invites further exploration. Up to now only few studies investigated the association between steroid hormones and social cognition in BPD and even less focused on testosterone. With regard to HPA axis function, results are heterogeneous and there is no evidence of a clear relationship between social cognition and cortisol release [4]. As described, the same is true for testosterone. While we reviewed some promising findings [26,27], more research is urgently needed.

There are multiple interconnections between hormonal systems such as the HPA and HPG axes, and focus on one single hormone might be too simplistic. Many other hormones and neuromodulators are involved in stress regulation and (social) cognition. For instance, the endocannabinoid system plays a role in the homeostatic regulation of the HPA axis [39]. In one of our own analyses (which was also motivated by Clemens Kirschbaum!), we found first evidence for lowered endocannabinoid levels in hair samples in BPD [40]. The oxytocin system is also important to consider, particularly with regard to social attachment, trust and social interactions. The above-mentioned study by Bonfig and colleagues observed a decrease in oxytocin in mothers with BPD during free-play with their children, which was not seen in healthy mothers [27]. Another study found that patients with BPD showed different oxytocin responses than healthy individuals after Cyberball, namely oxytocin reduction induced by social exclusion [41]. Other neurotransmitter systems like serotonin, dopamine and GABA are also possibly involved. All in all, further research is needed to take these complex interactions into account to better understand stress-related and social-interpersonal symptoms of BPD.

## 5 Testosterone and mental symptoms in BPD

Finally, the question arises whether testosterone levels are linked to mental symptoms. The most frequently reported finding is an association between low testosterone and depression, albeit the literature is not fully consistent. In women with BPD, a positive association between testosterone and depressive symptoms has been reported [23], which we were recently able to replicate in a larger sample [26]. Thus, evidence grows that in BPD depressive symptoms are related to increased testosterone, rather than lowered HPG axis activation. This also fits findings of Weber and colleagues, who reported elevated testosterone levels in women with severe MDD [16]. Furthermore, no associations between testosterone and impulsivity or aggression were found [23]. These results were surprising, as testosterone has been previously associated with aggression in women [17,30]. However, within the last years, evidence emerged that such association is not replicated in BPD [24,37]. It appears that testosterone might rather vary as a function of prosocial behavior, as described above.

It is important to note that BPD is a complex disorder with a high variety in symptomatology and it is likely that different endocrine features exist and subgroups differ significantly with respect to dominant psychopathology [42]. Several years ago, we suggested at least two subtypes: one predominantly characterized by trauma-associated, PTSD-like symptoms and another subgroup with mood disturbances as core symptoms [3], with both groups showing distinct HPA axis related alterations. However, gonadal hormones have not been investigated in this context yet. In addition to the positive relationship between testosterone and depressive symptoms we also found a positive correlation between testosterone and the severity of borderline symptoms measured with the borderline symptom list (BSL) [26]. The BSL [43] captures a wide range of symptoms within the BPD spectrum. In a further step we aimed to analyse whether any specific BPD symptoms were related to circulating testosterone levels. We also took the single DSM criteria of BPD into account and analyzed self-rated depressive symptoms, measured with the Beck's depression inventory (BDI-II) [44]. Results from both BSL-23 and BDI-II were highly consistent, showing a well-defined cluster of internalizing symptoms that correlated with baseline testosterone levels [45]. In particular, patients with higher testosterone levels showed higher ratings of self-dislike, feelings of senselessness, pessimism, and the feeling of being a failure. This resonates with previous findings from bipolar disorder, where higher testosterone levels have been associated with a higher number of previous suicide attempts [46].

The mechanism through which testosterone might influence mood-related symptoms is not clear and future research is required to address this question. One hypothesis focuses on amygdala function. The amygdala is rich in testosterone receptors and testosterone administration increases amygdala reactivity in young and middle-aged women [47,48]. Furthermore, the hippocampus and the prefrontal cortex are discussed to play in important role, particularly in mediating the effects of testosterone on (social) cognition [1]. The interplay between these brain areas might also modulate (mood-dependent) cognition about the self and social interaction with others.

## 6 Summary and conclusion

**Fig. 2:**
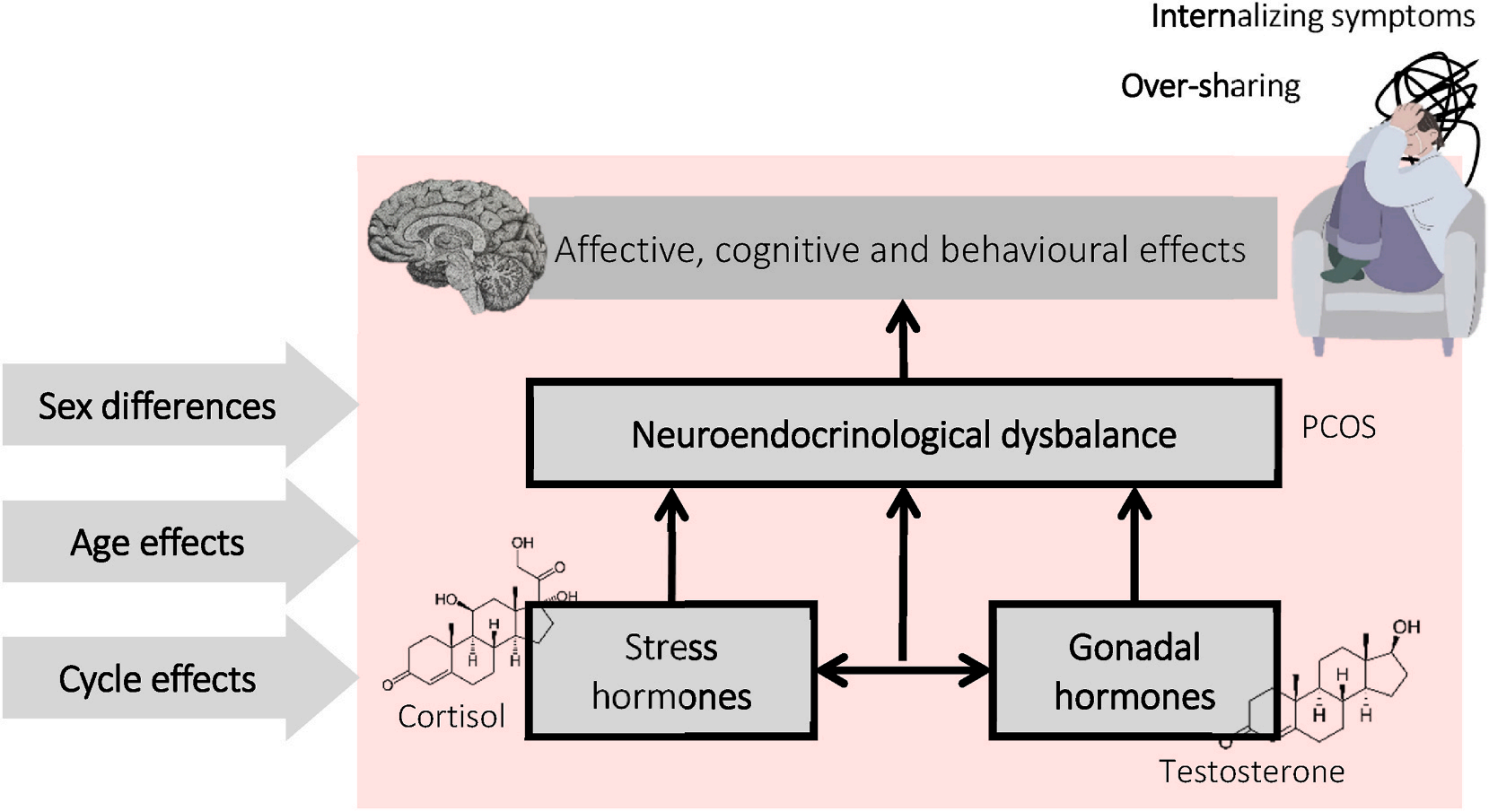
Visual summary.

In summary, converging evidence from hair, saliva, and serum analyses indicates that borderline personality disorder is characterized by altered HPG axis functioning, most consistently reflected in elevated basal testosterone levels in women. These alterations appear robust across samples and measurement modalities and are not accompanied by corresponding increases in long-term cortisol secretion, suggesting some level of dissociation between HPA and HPG axis activity in BPD. Importantly, testosterone elevations in this disorder do not map onto aggression or externalizing behaviour, but rather show associations with prosocial behaviour and internalizing symptoms such as depressive mood, self-devaluation, and pessimism. Together with findings regarding stress-related endocrine responses, these results support the notion that testosterone may play a modulatory role in social-affective functioning and symptom expression in BPD, potentially in interaction with cortisol and other neuromodulatory systems (see Fig. 2).

However, the relevance of age and sex-dependent factors, especially the female menstrual cycle, must be considered when interpreting these findings. Hormonal fluctuations related to the menstrual cycle can influence both testosterone and cortisol levels, meaning that sex differences and hormonal phases may contribute to variability in the clinical presentation of BPD, especially among females. Recent findings show that BPD symptoms in patients showed cycle-related fluctuations associated with fluctuating levels of estradiol and progesterone, in particular an exacerbation of symptoms during menses [49]. Furthermore, hormonal contraception further seems to affect BPD symptom severity [50]. In recent research, the notion of hormone sensitivity is receiving increased attention, that is the individual reactivity to both hormone levels and their fluctuations [51], which seems to be increased in BPD. The fact that the effects of hormones are not linear and vary between individuals makes it inevitable to consider not only between-group differences and absolute hormones levels, but extend research to within-group and within-individual associations throughout the menstrual cycle, the (reproductive) life-span and also the course of (clinical) interventions. This approach is promising for gaining a more fine-grained understanding of the interplay and dynamics between hormones and behaviour. Building on this accumulating evidence, future research should integrate age and sex-specific analyses and account for the cyclical hormonal changes in women to better understand their impact on BPD symptoms.

At the same time, the heterogeneity of BPD and the complexity of endocrine interactions caution against simplistic interpretations. As we argued throughout this paper, future research should adopt integrative, multimodal approaches that jointly consider HPA and HPG axis activity, additional hormonal and neuromodulatory pathways including the oxytocin and endocannabinoid system, and neurobiological mechanisms, ideally using longitudinal and symptom-focused designs. Such efforts, particularly when incorporating individual, sex and age-related variability, are urgently needed for a better understanding of biological mechanisms and potential subtypes within BPD symptomatology and thereby inform clinical interventions for stress regulation and social-interpersonal dysfunction in this disorder.

## CRediT authorship contribution statement

**Katja Wingenfeld:** Conceptualization. **Eugenia Kulakova:** Conceptualization.

## Declaration of competing interest

The authors declare that they have no competing interests.
